# Assessment of knowledge about childhood autism among paediatric and psychiatric nurses in Ebonyi state, Nigeria

**DOI:** 10.1186/1753-2000-5-1

**Published:** 2011-01-09

**Authors:** Monday N Igwe, Anthony C Ahanotu, Muideen O Bakare, Justin U Achor, Chinonyerem Igwe

**Affiliations:** 1 Department of Psychological Medicine, Ebonyi State University Teaching Hospital Abakaliki, Nigeria; 2 Child and Adolescent Unit, Federal Neuropsychiatric Hospital, New Haven, Enugu, Nigeria; 3 Drug Unit, Federal Neuropsychiatric Hospital, New Haven, Enugu, Nigeria

## Abstract

**Background:**

There is increasing public and professional awareness of autism spectrum disorders with early recognition, diagnosis and interventions that are known to improve prognosis. Poor knowledge about childhood autism among paediatric and psychiatric nurses who are members of multidisciplinary teams that care for such children may be a major barrier to early interventions that could improve quality of life and prognosis in childhood autism. Factors that influence knowledge about childhood autism among these nurses are not known. This study assessed knowledge about childhood autism among paediatric and psychiatric nurses in Ebonyi state, Nigeria and determined the factors that could be influencing such knowledge.

**Methods:**

Forty specialist paediatric and forty psychiatric nurses, making a total sample of eighty, were randomly selected from all the health care facilities in Ebonyi state, Nigeria. A socio-demographic questionnaire and knowledge about childhood autism among health workers (KCAHW) questionnaire were administered to them and the study was a point survey.

**Results:**

The total mean score on the KCAHW questionnaire among the nurses that participated in the study was 12.56 ± 3.23 out of a total of 19 possible. The mean score for the paediatric nurses was 11.78 ± 3.64 while psychiatric nurses had mean score of 13.35 ± 2.58. The mean scores in Domain 1 were 6.17 ± 1.75 for the paediatric nurses and 6.52 ± 1.43 for the psychiatric nurses. The mean scores in Domain 2 were 0.65 ± 0.48 for the paediatric nurses and 0.80 ± 0.41 for the psychiatric nurses. Domain 3 showed mean scores of 1.97 ± 1.25 for the paediatric nurses while psychiatric nurses scored 2.62 ± 1.23. Domain 4 yielded the mean scores of 2.97 ± 1.54 and 3.42 ± 0.98 for the paediatric and psychiatric nurses respectively.

There was significant relationship between the total mean score on the KCAHW questionnaire for the two groups and the area of specialisation of the nurses (t = -2.23, df = 78, p = 0.03) and there was also significant relationship between previous involvement in managing children with childhood autism as a specialist paediatric or psychiatric nurse and the total mean score on the KCAHW questionnaire (t = 6.90, df = 78, p = 0.00).

**Conclusion:**

The scores reflect deficits in knowledge about childhood autism among the study cohorts. Paediatric and psychiatric nurses as members of multidisciplinary teams that care for children with childhood autism are expected to provide holistic care and adequate counselling to the families of these children. Unfortunately in this environment, they are not fully equipped with enough knowledge about childhood autism. Education on childhood autism is therefore needed and can be provided through continuing medical education and emphasizing childhood autism in their training curriculum. This will enhance early identification and diagnosis of childhood autism with early interventions that are known to improve prognosis.

## Background

Childhood autism is a pervasive developmental disorder that affects children's social, communication and behavioural development. There are qualitative impairments in social interaction, communication with restricted repetitive and stereotyped patterns of behaviour, interests and activities [1]. Knowledge and awareness about this condition have been on the increase especially in the developed countries whereas these remain at a lower ebb in Nigeria and other sub-Saharan African countries [2,3]. Lack of knowledge and awareness about childhood autism is thus a major barrier to improving the health and wellbeing of children affected by autism in this environment. This further limits access to care and early interventions that are known to improve quality of life and prognosis in children with childhood autism.

Bakare et al [4] had noted that nurses working in tertiary health institutions in south - east and south - south regions of Nigeria scored low on the knowledge about childhood autism among health workers (KCAHW) questionnaire and knowledge gap was found to be higher in domain 3 (symptoms of obsessive and repeated pattern of behaviour), followed by domain 1 (symptoms of impairments in social interaction), domain 4 (type of disorder childhood autism is and associated co-morbidity) and domain 2 (symptoms of communication impairments). Knowledge about childhood autism was also significantly associated with older age groups, previous experience managing children with autism spectrum disorders, years of experience as a health worker and area of specialty with workers in psychiatric facility scoring higher than those working in paediatric settings.

Another survey showed that the majority of school nurses are knowledgeable about autism spectrum disorders, including symptomatology and related medications but are not as knowledgeable concerning communication skills, behavioural therapies and safety issues [5].

Igwe et al [6] reported that undergraduate medical students had higher scores on the KCAHW questionnaire followed by undergraduate nursing and psychology students scoring the least. They advocated for additional exposure of the undergraduate psychology students to training curriculum aimed at improving their early recognition of symptoms and signs of autism spectrum disorders which is known to improve prognosis.

Earlier on Shah had assessed the awareness about childhood autism among two hundred and fifty medical students at different stages of their training. He compared differences between first-year and fourth-year students with respect to their knowledge of various aspects of autism, including diagnosis, cause, symptomatology, treatment and outcome. Fourth-year students were only significantly more likely to respond correctly to questions related to diagnostic criteria and core symptoms. His findings suggest that more emphasis should be placed on teaching medical students about childhood autism to enhance early diagnosis and interventions [7].

To assess individual's beliefs and knowledge about childhood autism, Stone developed the Autism Survey. This has been used to compare knowledge and beliefs of individuals from different professional philosophies, teachers and parents about autism [8]. Stone subsequently used the survey to compare the views of paediatricians, clinical psychologists, school psychologists, speech and language pathologists and autism specialists. Results indicated that individual disciplines studied displayed variations and historic misconceptions regarding social, emotional and cognitive aspects of autism. However the autism specialists viewed the cognitive abilities of individuals with autism more realistically than other professionals in the study [9].

In another study that compared the views of parents of people with autism, teachers and autism specialists, it was found that parents held some beliefs about autism not shared by teachers and autism specialists. Parents were more likely than teachers and autism specialists to believe that autism was a temporary condition and that with time the children will overgrow it. Also the parents overestimated the cognitive abilities of children with autism [10].

Greek teachers have been observed to know more about learning disabilities followed by autism and attention deficit/hyperactivity disorder irrespective of whether they were special needs or general teachers. The author called for specialised training of teachers on special education needs [11].

Poor knowledge of autism spectrum disorders among physicians and failure to give further information to caregivers may be a reflection of lack of training in the wide range of behaviours that occur across the autism spectrum. This may delay average age of diagnosis and subsequently early interventions that are established to be beneficial [12].

Caring for children with childhood autism and other pervasive developmental disorders requires the services of professionals like psychiatrists, paediatricians, nurses, clinical psychologists among others [13]. Paediatric and psychiatric nurses are usually members of such multidisciplinary teams.

The most significant role of a nurse in autism recognition and diagnosis is education. The nurse, the family, and the patient must all be educated on various aspects of autism and autistic disorders. This places nurses at a critical juncture, because they must be increasingly knowledgeable, understanding and supportive of the parents and children afflicted with this condition. The nurse's level of understanding of autism spectrum disorders can have a great impact on the prognosis of children with childhood autism.

However, a study found that only ten percent of parents had their child's condition explained to them in a clinical setting [14]. It is uncertain whether this could be a reflection of poor knowledge and awareness about childhood autism among paediatric and psychiatric nurses engaged in caring for children with autism.

Therefore, there is the need to assess the level of knowledge about childhood autism among paediatric and psychiatric nurses who are usually members of the multidisciplinary team that care for children with autism and who also act as educators and advocates for this group of children. This study is aimed at assessing base line knowledge about childhood autism and evaluating factors that influence such knowledge among paediatric and psychiatric nurses in Ebonyi State, Nigeria

## Methods

### Location

Location of the study was Ebonyi State, Nigeria. Ebonyi State is a mainland south-eastern state of Nigeria, inhabited and populated primarily by the Igbo ethnic group. It is one of the 36 states in Nigeria and one of the 5 states in the south-eastern geopolitical zone of the country. South-east is one of the 6 geopolitical zones in Nigeria. Ebonyi state was created in 1996 from the old Abakaliki division of Enugu state and old Afikpo division of Abia state with the capital sited at Abakaliki which is also the largest city. Ebonyi is primarily an agricultural producing region but also has several solid mineral resources including huge salt deposits at Uburu and Okposi, hence it is called 'The salt of the Nation.' There are 13 General Hospitals, one located in each of the 13 local government areas. The Federal Medical Centre and a State Teaching Hospital, which are tertiary health facilities, are located at Abakaliki. There are also many mission and private hospitals within the state.

### Ethical approval

The ethical approval for the study was obtained from the Institutional Review Board (IRB) of Ebonyi State University Teaching Hospital Abakaliki, Ebonyi State Nigeria. Written informed consent was also obtained from the respondents that participated in the study.

### Participants and sampling method

The participants involved in this study were paediatric and psychiatric nurses who work in health facilities spread across Ebonyi State. Each had already obtained a diploma in paediatric or psychiatric nursing in addition to a registered nurse certificate. There are about fifty registered psychiatric nurses and forty three paediatric nurses working in Ebonyi state, Nigeria. Forty nurses were randomly selected from each group making a total sample size of eighty. The study was a point survey.

## Materials

### Socio-demographic questionnaire

A socio-demographic questionnaire was used to obtain information like gender, age, marital status, ethnicity, duration of working experience as a specialist nurse and previous experience managing children with childhood autism.

### Knowledge about childhood autism among health workers (KCAHW) questionnaire [2]

This is a self-administered questionnaire that was developed by a team of psychiatrists and clinical psychologists in 2008 at Enugu, Nigeria. It contains a total of nineteen questions. The KCAHW questionnaire has been used in several studies and has been established to have good test-retest reliability, good overall internal consistency (cronbach's alpha value of 0.97) and culturally valid [2]. It is used to assess baseline knowledge about childhood autism among the health workers. Each of the nineteen items has three options to choose from with only one out of the three being correct. The correct option on each item attracts a score of 1, whereas the other two incorrect options are scored 0 each.

The KCAHW questionnaire is divided into the following four domains:

#### Domain 1

This domain contains eight items that address the impairments in social interaction usually found in children with childhood autism. A maximum score of 8 and minimum score 0 are possible in this domain.

#### Domain 2

This domain contains only one item that addresses impairment in the area of communication and language development, as part of the symptoms seen in children with childhood autism. A maximum score of 1 and minimum score of 0 are possible in this domain.

#### Domain 3

This domain contains four items that address the area of obsessive and compulsive pattern of behaviour found in children with childhood autism, a pattern of behaviour which had been described as restricted, repetitive and stereotyped. A maximum score of 4 and minimum score of 0 are possible in this domain.

#### Domain 4

This domain contains six items that address knowledge on what type of disorder childhood autism is, possible co-morbid conditions and period of onset of childhood autism in affected children. A maximum score of 6 and minimum score 0 are possible in this domain.

A maximum total score of 19 and minimum total score of 0 are possible when the four domain scores are summed up. The questionnaire and the scoring system are shown in Appendix 1. The mean total score on the KCAHW questionnaire among a particular sample population is a measure of level of knowledge about childhood autism among that particular population. A total score of 19, which is the maximum score possible on the KCAHW questionnaire, indicates adequate knowledge of symptoms and signs of autism. This adequate knowledge may enhance early recognition, diagnosis, appropriate referral and interventions that are known to improve prognosis in children with childhood autism.

### Procedure

The socio-demographic and KCAHW questionnaires were administered to the eighty (forty paediatric and forty psychiatric) nurses. The questionnaires were completed by the respondents and collected back from them at the point of administration to prevent them from consulting study materials or discussing with colleagues before filling their responses.

### Data analysis

The generated data were analysed using Statistical Package for Social Sciences (SPSS) version 16. The mean score in each domain and the mean total score were calculated for the two groups of nurses. The mean total score were related to the socio-demographic variables of the respondents using independent sample t-test.

## Results

A total of eighty (80) nurses consented to participate in the study, forty being paediatric nurses and forty psychiatric nurses. There were five (12.5%) male and thirty five (87.5%) female paediatric nurses while nineteen (47.5%) male and twenty one (52.5%) female were psychiatric nurses. The mean age of the paediatric nurses was 33.95 ± 7.89 years and 37.25 ± 7.32 years for the psychiatric nurses. Nine (22.5%) paediatric nurses had previous experience nursing children with childhood autism while thirty one of them (77.5%) had not been involved in managing children with autism. Eighteen psychiatric nurse (45%) have participated in managing children with childhood autism while twenty two (55%) had previously not been involved. Other socio-demographic variables of the participants are shown in Table 1.

**Table 1:** Socio-demographic variables of the nurses

Socio-demographic		
variables	Paediatric nurses	Psychiatric nurses
**Gender**		
Male	5 (12.5%)	19 (47.5%)
Female	35 (87.5%)	21 (52.5%)

**Age (years)**		
Mean ± SD	33.95 ± 7.89	37.25 ± 7.32

**Marital status**		
Single	16 (40%)	13 (32.5%)
Married	22 (55%)	27 (67.5%)
Divorced/separated	0 (0%)	0 (0%)
Widowed	2 (5%)	0 (0%)

**Ethnic group**		
Igbo	38 (95%)	40 (100%)
Yoruba	1 (2.5%)	0 (0%)
Others	1 (2.5%)	0 (0%)

**Religion**		
Christianity	40 (100%)	37 (92.5%)
Islam	0 (0%)	2 (5%)
Traditional	0 (0%)	1 (2.5)

**Duration of work as specialist nurse (yrs)**	5.9 ± 5.5	7.9 ± 5.2

**Previous experience nursing children with autism**	9 (22.5%)	18 (45)

### Pattern of distribution of scores on the KCAHW questionnaire among the nurses

Maximum possible score on the knowledge about childhood autism among health workers (KCAHW) questionnaire is nineteen (19) and a minimum score of zero (0). The questionnaire is divided into domains 1, 2, 3 and 4 with maximum possible scores of 8, 1, 4 and 6 respectively. A minimum score of zero (0) is possible in each of the four domains [2]. The total mean score on the KCAHW questionnaire among the nurses that participated in the study was 12.56 ± 3.23 out of a total of 19 possible. The mean score for the paediatric nurses was 11.78 ± 3.64 while psychiatric nurses had mean score of 13.35 ± 2.58. The mean total scores in Domain 1, which is concerned with questions in the area of impairments in social interaction as found in childhood autism, were 6.17 ± 1.75 for the paediatric nurses and 6.52 ± 1.43 for the psychiatric nurses. The mean total scores in Domain 2 which addresses communication impairments that often characterized childhood autism were 0.65 ± 0.48 for the paediatric nurses and 0.80 ± 0.41 for the psychiatric nurses. Domain 3, which deals with questions on obsessive and repetitive behavioural patterns that are often seen in childhood autism, showed total mean scores of 1.97 ± 1.25 for the paediatric nurses while psychiatric nurses scored 2.62 ± 1.23. Domain 4 that covers questions on what type of disorder childhood autism is and possible associated co-morbidity yielded the total mean scores of 2.97 ± 1.54 and 3.42 ± 0.98 for the paediatric and psychiatric nurses respectively.

Psychiatric nurses who have had experience of nursing children with autism scored 15.35 ± 0.86 on the KCAHW questionnaire while their paediatric colleagues scored 15.30 ± 1.89. Those who have not had experience of nursing children with autism scored 10.60 ± 3.32 and 11.87 ± 2.42 for paediatric and psychiatric nurses respectively.

The mean scores in domains 1, 2, 3, 4 and total mean score are higher among the psychiatric nurses than the paediatric nurses indicating that the psychiatric nurses are more likely to recognise symptoms and signs of autism than the paediatric nurses. The pattern of distribution of scores on the KCAHW questionnaire is shown in Table 2.

**Table 2:** Pattern of distribution of scores on the KCAHW questionnaire among the nurses.

Domains Possible score		Paediatric nurses	Psychiatric nurses
**Domain 1**	8	6.17 ± 1.75	6.52 ± 1.43

**Domain 2**	1	0.65 ± 0.48	0.80 ± 0.41

**Domain 3**	4	1.97 ± 1.25	2.62 ± 1.23

**Domain 4**	6	2.97 ± 1.54	3.42 ± 0.98

**Mean score by nurses with experience of autism**	19	15.30 ± 1.89	15.35 ± 0.86

**Mean score by nurses without experience of autism**	19	10.60 ± 3.32	11.87 ± 2.42

**Total mean score**	19	11.78 ± 3.64	13.35 ± 2.58

### Factors affecting knowledge about childhood autism among the nurses

There was significant relationship between the total mean score on the KCAHW questionnaire and the area of specialisation of the nurses (t = -2.23, df = 78, p = 0.03). The paediatric nurses scored 11.78 ± 3.64 as against mean score of 13.35 ± 2.58 by the psychiatric nurses. The psychiatric nurses also scored higher than the paediatric nurses in all the four domains.

Significant relationship was also found between total mean score on the KCAHW questionnaire and previous experience nursing children with childhood autism as a specialist paediatric or psychiatric nurse (t = 6.90, df = 78, p = 0.00).

Greater number of psychiatric nurses had experience in nursing children diagnosed as having childhood autism compared to paediatric nurses. Eighteen psychiatric nurses (45%) had such experience while only nine (22.5%) of paediatric nurses have been in contact with children who have childhood autism. The mean score for paediatric nurses who had cared for children with autism was 15.30 ± 1.89 while the psychiatric nurses who have had such experience scored 15.35 ± 0.86 on the KCAHW questionnaire.

There was no significant relationship between age and total mean score on the KCAHW questionnaire among the two groups. The mean age of the paediatric nurses was 33.95 ± 7.89 years and 37.25 ± 7.32 years for the psychiatric nurses. There is no significant difference in the mean ages of the two groups (t = -1.94, df = 78, p = 0.06). No significant relationship was also found between the total mean score on the KCAHW questionnaire and duration of work as a specialist nurse (t = -1.66, df = 78, p = 0.10).

## Discussion

The total mean score among the two groups of nurses studied was 12.56 ± 3.23 out of possible score of 19. This is not significantly different from the score of 12.35 ± 4.40 obtained among practising nurses in our earlier study [4].

Area of specialisation was a factor that influenced knowledge about childhood autism with psychiatric nurses scoring higher than the paediatric nurses on the KCAHW questionnaire. Psychiatric nurses are more likely to recognise symptoms and signs of childhood autism compared to paediatric nurses and this will certainly aid early recognition, diagnosis with prompt interventions that are known to improve prognosis for children with childhood autism.

Significant relationship also existed between previous experience managing children with childhood autism and scores on the KCAHW questionnaire with those who said yes to having nursed such children scoring higher. Children with childhood autism in this environment are more likely to be brought to psychiatric facilities rather than paediatric facilities probably because of the associated behavioural problems, epilepsy and learning disability [4]. Hence psychiatric nurses are more likely to come in contact with children with childhood autism in this environment.

The variation in knowledge about childhood autism as seen in this study concurs with significant variations between specialists in different health care settings involved in caring for children with autism [4,6,9,15].

No significant association was found between knowledge about childhood autism and number of years of work as a specialist nurse. There was also no significant relationship between age of the nurses and scores on the KCAHW questionnaire. Those who are older and probably with longer years of experience may not necessarily score higher on the KCAHW questionnaire. This may not be unconnected with recent upsurge in awareness and research in autistic spectrum disorders [16-18]. This is in contrast with a previous finding which observed that knowledge about childhood autism was higher in nurses with 6 to 20 years working experience and those who are in their fourth decade of life and above [4].

Childhood neuro-developmental disorders are increasingly being recognised with high demands for earlier diagnosis and intervention. However the total mean score of 12.56 ± 3.23 out of a total of 19 possible on the KCAHW questionnaire by the study group is low and is a reflection of deficits in knowledge, education and awareness about childhood autism among the paediatric and psychiatric nurses in this environment.

### Limitations of the study

The KCAHW questionnaire is fashioned to be self administered and collected immediately. This is aimed at avoiding consulting study materials or discussing with other health workers which may influence the responses of the subjects to questions contained in the questionnaire. Thus the KCAHW questionnaire only gives a point assessment of knowledge. The questionnaire also does not assess etiological explanations and other cultural beliefs held by the respondents about childhood autism.

## Conclusion

The scores reflect deficits in knowledge about childhood autism among the study cohorts. Paediatric and psychiatric nurses as members of multidisciplinary teams that care for children with childhood autism are expected to provide holistic care and adequate counselling to the families of these children. Unfortunately in this environment, they are not fully equipped with enough knowledge about childhood autism. Education on childhood autism is therefore needed and can be provided through continuing medical education and emphasizing childhood autism in their training curriculum. This will enhance early identification and diagnosis of childhood autism with early interventions that are known to improve prognosis.

## Appendix 1

### Knowledge about Childhood Autism among Health Workers (KCAHW) Questionnaire

Please do not consult formal text books to answer these questions.

Thank you for your co-operation.

The following behaviours best describe a child with childhood autism:

#### Domain 1

i. Marked impairment in use of multiple non-verbal behaviours such as eye to eye contact, facial expression, body postures and gestures during social interaction?

(A) Don't Know, (B) Yes, (C) No

ii. Failure to develop peer relationship appropriate for developmental age?

(A) Don't Know, (B) Yes, (C) No

iii. Lack of spontaneous will to share enjoyment, interest or activities with other people? (A) Don't Know, (B) Yes (C) No

iv. Lack of social or emotional reciprocity? (A) Don't Know (B) Yes, (C) No

v. Staring into open space and not focusing on anything specific? (A)Don't Know, (B) Yes, (C) No

vi. The child can appear as if deaf or dumb? (A) Don't Know (B) Yes, (C) No

vii. Loss of interest in the environment and surroundings?

(A) Don't Know, (B) Yes, (C) No

viii. Social smile is usually absent in a child with Autism?

(A)Don't Know, (B) Yes (C) No

#### Domain 2

i. Delay or total lack of development of spoken language?

(A) Don't Know (B) Yes (C) No

#### Domain 3

i. Stereotyped and repetitive movement (e.g. Hand or finger flapping or twisting)?

(A) Don't Know (B) Yes, (C) No

ii. May be associated with abnormal eating habit?

(A) Don't Know, (B) Yes, (C) No

iii. Persistent preoccupation with parts of objects?

(A) Don't Know (B) Yes, (C) No

iv. Love for regimented routine activities? (A) Don't Know (B) Yes, (C) No

#### Domain 4

i. Autism is Childhood Schizophrenia? (A) Don't Know (B) Yes (C) No

ii. Autism is an auto-immune condition? (A) Don't Know (B) Yes (C) No

iii. Autism is a neuro-developmental disorder? (A) Don't Know (B) Yes (C) No

iv. Autism could be associated with Mental Retardation? (A) Don't Know (B) Yes (C) No

v. Autism could be associated with Epilepsy? (A) Don't Know (B) Yes (C) No

vi. Onset of Autism is usually in, (A) Neonatal age, (B) Infancy, (C) Childhood

### Scoring of Knowledge about Childhood Autism among Health Workers (KCAHW) questionnaire

#### Domain 1

i Marked impairment in use of multiple non-verbal behaviours such as eye to eye contact, facial expression, body postures and gestures during social interaction?

(A) 0 (B) 1 (C) 0

ii Failure to develop peer relationship appropriate for developmental age? (A) 0 (B) 1 (C) 0

iii. Lack of spontaneous will to share enjoyment, interest or activities with other people?

(A) 0 (B) 1 (C) 0

iv Lack of social or emotional reciprocity? (A) 0 (B) 1 (C)

v Starring into open space and not focusing on anything specific?

(A) 0 (B) 1 (C) 0

vi. The child can appear as if deaf or dumb? (A) 0 (B) 1 (C) 0

vii. Loss of interest in the environment and surroundings? (A) 0 (B) 1 (C) 0

viii. Social smile is usually absent in a child with Autism? (A) 0 (B) 1 (C) 0

#### Domain 2

i. Delay or total lack of development of spoken language? (A) 0 (B) 1 (C) 0

#### Domain 3

i. Stereotyped and repetitive movement (e.g. Hand or finger flapping or twisting)?

(A) 0 (B) 1 (C) 0

ii. May be associated with abnormal eating habit? (A) 0 (B) 1 (C) 0

iii. Persistent preoccupation with parts of objects? (A) 0 (B) 1 (C) 0

iv. Love for regimented routine activities? (A) 0 (B) 1 (C) 0

#### Domain 4

i. Autism is Childhood Schizophrenia? (A) 0 (B) 0 (C) 1

ii. Autism is an auto-immune condition? (A) 0 (B) 0 (C) 1

iii Autism is a neuro-developmental disorder? (A) 0 (B) 1 (C) 0

iv. Autism could be associated with Mental Retardation? (A) 0 (B) 1 (C) 0

v. Autism could be associated with Epilepsy? (A) 0 (B) 1 (C) 0

vi Onset of Autism is usually in, (A) 0 (B) 0 (C) 1

A total maximum score of 19 and a minimum score of 0 are possible. The average score on the KCAHW questionnaire among a particular sample population gives an index level of knowledge about childhood autism in that particular population.

## Competing interests

The authors declare that they have no competing interests.

## Authors' contributions

CI was involved in collection of data while MNI was involved in writing the initial draft of the manuscript and data analysis. All the authors contributed to the conception of the study and were involved in writing and revising the manuscript. All the authors read and approved the final draft of the manuscript.
